# Comparison of Newborn Hearing Screening in Well-Baby Nursery and NICU: A Study Applied to Reduce Referral Rate in NICU

**DOI:** 10.1371/journal.pone.0152028

**Published:** 2016-03-29

**Authors:** Pei-Chun Li, Wei-I Chen, Chih-Ming Huang, Ching-Ju Liu, Hsiu-wen Chang, Hung-Ching Lin

**Affiliations:** 1 Department of Audiology and Speech Language Pathology, Mackay Medical College, Taipei, Taiwan; 2 Department of Speech and Hearing Disorders and Sciences, National Taipei College of Nursing, Taipei, Taiwan; 3 Department of Otolaryngology, Mackay Memorial Hospital, Taitung, Taiwan; 4 Department of Otolaryngology, Mackay Memorial Hospital, Taipei, Taiwan; 5 Department of Medicine, Mackay Medical College, Taipei, Taiwan; Hôpital Robert Debré, FRANCE

## Abstract

**Objectives:**

To determine whether newborn hearing screening in a well-baby nursery (WBN) and neonatal intensive care unit (NICU) nursery: 1) meet three targeted, screening, referral, and diagnostic follow-up rates; 2) compare the average age of diagnosis for infants admitted to the WIN and NICU; and 3) determine prevalence of hearing loss in neonatal population; and 4) try to find a practical newborn hearing screening time algorithm to reduce refer rate in NICU

**Materials and Methods:**

It examined 15,624 newborns in the WBN (13,676) and NICU (1948) screened for congenital HL using AABR. The variables analyzed in it were the screening rate, referral rate, follow-up rate, diagnostic rate and diagnostic age, prevalence rate, degrees of congenital bilateral HL. The study was approved by the hospital’s institutional review board (13MMHISO23).

**Results:**

The screening rates were 99.8% and 99.6% in the WBN and NICU groups, respectively, without significant difference. The referral rates were 0.7% and 2.8% in the WBN and NICU groups, with significant difference. Furthermore, the diagnostic follow-up rates were 76.7% and 89.1% in the WBN and NICU groups, without significant difference. The average initial diagnostic ages were 1.9 months and 3.8 months in the WBN and NICU groups, with significant difference. The prevalence of congenital bilateral hearing loss were 0.27% and 1.6% in the WBN and NICU groups, with significant difference.

**Conclusion:**

The screening, referral and follow-up rate in the WBN and NICU groups were equivalent to the quality indicators. For NICU group, screening and diagnostic follow up were performed later than those in WBN group; however the lower referral rate in our NICU group was successfully achieved in this study and can be applied clinically. The prevalence of congenital bilateral hearing loss was higher in the NICU group than in the WBN group.

## Introduction

The importance of universal early screening, diagnosis, and intervention in reducing the negative impact of congenital hearing loss (HL) has been described extensively all over the world[1–5]. Mackay Memorial Hospital and the Children’s Hearing Foundation established the first pilot hospital-based program for newborn hearing screening in Taiwan in 1998^4^. During 2003–2013, the following three major initiatives supported by the Bureau of Health Promotion were introduced to promote UNHS in Taiwan: (1) the establishment of “2004 The Guidance of Newborn Hearing Screening” (using otoacoustic emissions [OAE] or automated auditory brainstem response [AABR] examination, the expense of which was to be borne by the parents); (2) the establishment of “2008 Taiwan Newborn Hearing Screening Consensus Document” (using AABR examination, the expense of which was to be borne by the parents), which has three major goals, namely achieving a screening rate of >95%, a referral rate of <4%, and a diagnostic follow-up rate of >70%[6]; and (3) the establishment of “2014 Taiwan Newborn Hearing Screening Consensus Document”(a screening rate of >95%, a referral rate of < 2%, and a diagnostic follow-up rate of > 95%)[7] and the implementation of a free national UNHS program UNHS in 2012 (using only AABR examination to concisely reduce false-positive and false-negative findings; the costs of AABR examination are borne by the government, and the examination is therefore free of cost for parents). The age of identification and diagnosis of as well as interventions for congenital hearing-impaired children have decreased gradually since the government established the newborn hearing screening program in Taiwan[5].

In Taiwan, the national program for UNHS by using AABR examination, free of cost for citizens, was implemented in 2012 and then every Taiwanese newborn baby, including those in the well-baby nursery (WBN) and neonatal intensive care unit (NICU), should receive hearing screening before discharge from the hospital. In our previous studies[8–11], all babies who were screened for hearing impairment were from the WBN, not from the NICU, and were healthy. No study of UNHS in NICU babies has been conducted in Taiwan. In 2000, the U.S. Joint Committee on Infant Hearing recommended that NICU babies undergo hearing screening tests[12]. Connolly et al[13] reported that the incidence of congenital HL according to the high risk register was 1.2/1000 in BR babies and 13.3/1000 in NICU babies. Moreover, in one study, the incidence of auditory neuropathy spectrum disorder (ANSD) in NICU babies was 24%, and screening them by using OAE examination resulted in false-negative findings[14]. In another study, OAE examination was affected by the environmental noise level in the NICU, which is usually higher than 60 dBA according to a statement issued by the ASHA[15]. Moreover, the U.S. NIH (1993) recommended that AABR, not OAE, examination be used as a hearing screening technique for babies with a high risk of HL according to the high risk register [16].

Study of UNHS in NICU babies has not been conducted before in Taiwan and several in other countries[17–19], however which showed much higher refer rate in NICU in U.S. of 4.9%[17], Dutch of 9.2%[18] and Brazil of 4.1%[19]. So, the aims of study were to assess whether the screening babies in the WBN and NICU using AABR enables achieving the three aforementioned goals[6–7], namely a screening rate >95%; a referral rate <4%; and a diagnostic follow-up rate >70% and compared the average age of congenital HL diagnosis as well as the prevalence of congenital HL between WBN and NICU babies. Finally, we try to find a practical newborn hearing screening time algorithm to reasonably reduce refer rate in NICU to prevent burden of audiology follow up and intangible cost including parental anxiety.

## Materials and Methods

This retrospective cohort study examined well-baby nursery (WBN) and NICU babies at Mackay Memorial Hospital, Taipei screened between February 2008 and December 2011 and was approved by the hospital’s institutional review board (13MMHISO23) with chairman of Yen-Ta Lu M.D. PhD. All patients records/information were anonymized and de-identified prior to analysis. Mackay Memorial Hospital, Taipei is a big medical center in northern Taiwan, the birth babies come from more than 1/4 of total country population, and is the epitome of newborn hearing health care in Taiwan. A total of 15,624 newborns (WBN, 13,676; NICU, 1948) underwent screening for congenital HL through AABR examination (ALGO 3i, Natus Medical Inc. San Carlos, CA USA). There was none with craniofacial anomaly in WBN group. Two experienced (> 10 years work) full-time nurses were involved in the initial hearing screening program. In Taiwan, the medical care system allows only 3 days postnatal hospitalization for a healthy newborn. The appropriate time for performing hearing screening is considered to be 48 hours postnatal. NICU babies with several health problems and hearing impairment are easily affected by environmental noise in the NICU. Consequently, screening NICU babies for HL is more difficult than screening WBN babies; NICU babies undergo hearing screening after their condition stabilizes and they are moved to the newborn center (NBC). Babies fail to pass initial AABR screening undergo a second AABR screening before their discharge. However, if they fail to pass the second AABR screening, they should undergo complete hearing diagnostic assessment.

In this study, the initial diagnostic hearing tests were TEOAE, click-Auditory Brainstem Response, and tympanometry. For babies aged 1 month who required further assessment, diagnostic ABR testing was performed using an Air Conduction click stimulus. Babies were considered to have sufficient hearing sensitivity for the development of speech and language if bilateral ABR threshold searching at 20 dB normalized hearing level (dB nHL) showed the presence of a Wave V. Babies were considered to have normal hearing if they fulfilled the pass criteria for both TEOAE and ABR examinations. For babies who failed to pass the initial ABR or OAE examination, a second diagnostic hearing test involving tone-burst ABR measurement (500, 1000, 2000, and 4000 Hz), Auditory Steady State Response, and tympanogram was conducted.

Babies who failed to pass the second stage of tone-burst ABR or ASSR testing received behavioral hearing tests (including behavior observation audiometry for those aged < 6 months and visual reinforcement audiometry or those aged 6–30 months). HL at a VRA or ABR threshold of 25–39 dB nHL was defined as mild, HL at a threshold of 40–69 dB nHL was considered moderate, HL at a threshold of 70–89 dB nHL was defined as severe, and HL at a threshold of ≥90 dB nHL was considered profound. HL was defined as bilateral asymmetrical when the difference in the hearing threshold between the ears was >15 dB HL, and the degree of bilateral asymmetrical HL depended on the ear with milder HL. Parent counseling and fitting of the hearing aid were arranged for the babies with confirmed bilateral HL.

SPSS 19.0 and the chi-square test were used to analyze and compare the hearing screening performance between the WBN and NICU babies. The independent *t* test was used to compare the screening age and diagnostic age of HL.

## Results

Between February 2008 and December 2011, a total of 15,624 newborns (WBN, 13,676; NICU, 1948) were screened for HL at Mackay Memorial Hospital (a tertiary medical center), Taipei, Taiwan. Of the 13,676 babies in the WBN group, 13,645 were screened for HL, representing a hearing screening coverage rate of 99.8%; of the 1948 babies in the NICU group, 1941 were screened for HL, representing a hearing screening coverage rate of 99.6%. The chi-square test showed no significant difference between the groups (*X*^2^ = 1.237 (df = 1, p = 0.266), p > 0.01). Of the 13,676 babies in the WBN group and 1948 babies in the NICU group, 13,603 and 1734 underwent early screening (before 1 month), accounting for rates of 99.7% and 89.3%, respectively. The chi-square test showed a significant difference between the groups (*X*^2^ = 1183.735 (df = 1, p = 0.000), p < 0.01). The average ages of hearing screening were 2.12(±0.5) days and 13.05(±2.6) days in the WBN and NICU groups, respectively. There was a significant difference between the two groups (F-test = 4841.990 (p = 0.000), t = −22.489 (df = 1958.292,p = 0.000), p < 0.01). It meant NICU babies received later hearing screening due to unhealthy state.

**Fig 1:**
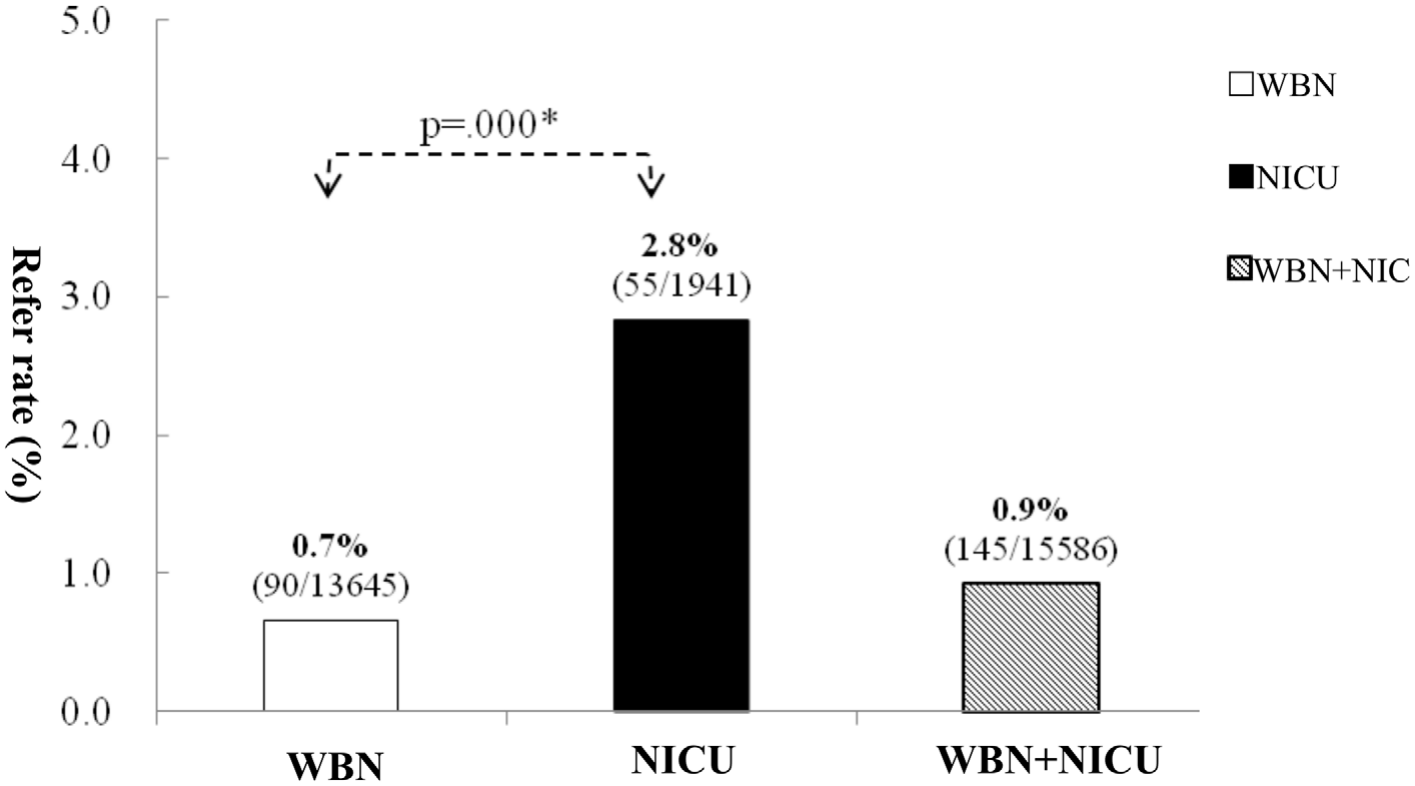
Referral rate at discharge of babies in the WBN and NICU groups. The chi-square test showed a significant difference of referral rate between the BR and NICU groups (X2 = 87.139 (df = 1, p = 0.000), p < 0.01).

Fig 1 shows that the total hearing screening referral rate was 0.9% (0.7% and 2.8% in the WBN and NICU groups, respectively). The chi-square test showed a significant difference of referral rate between the WBN and NICU groups (*X*^2^ = 87.139 (df = 1, p = 0.000), p < 0.01).

**Fig 2:**
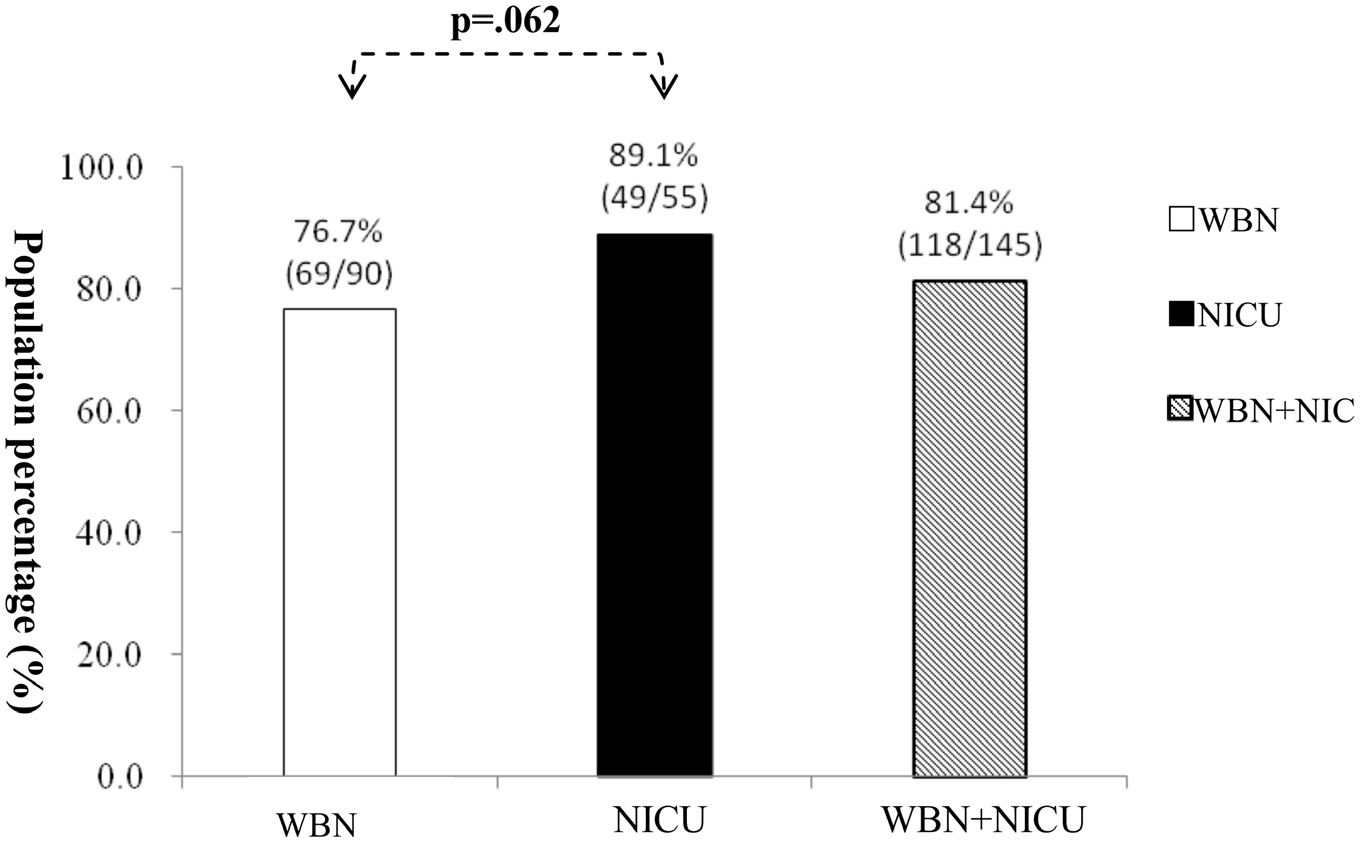
OPD return rate of WBN (n = 90) and NICU (n = 55). There was no significant difference between the BR and NICU groups (X2 = 3.478 (df = 1, p = 0.062), p > 0.01)babies.

The overall return-for-follow-up rate after failure of hearing screening in both groups was 81.4% (118/145). Fig 2 shows the rates to be 76.7% (69/90) and 89.1% (49/55) in the WBN and NICU groups, respectively. There was no significant difference between the WBN and NICU groups (*X*^2^ = 3.478 (df = 1, p = 0.062), p > 0.01).

**Fig 3:**
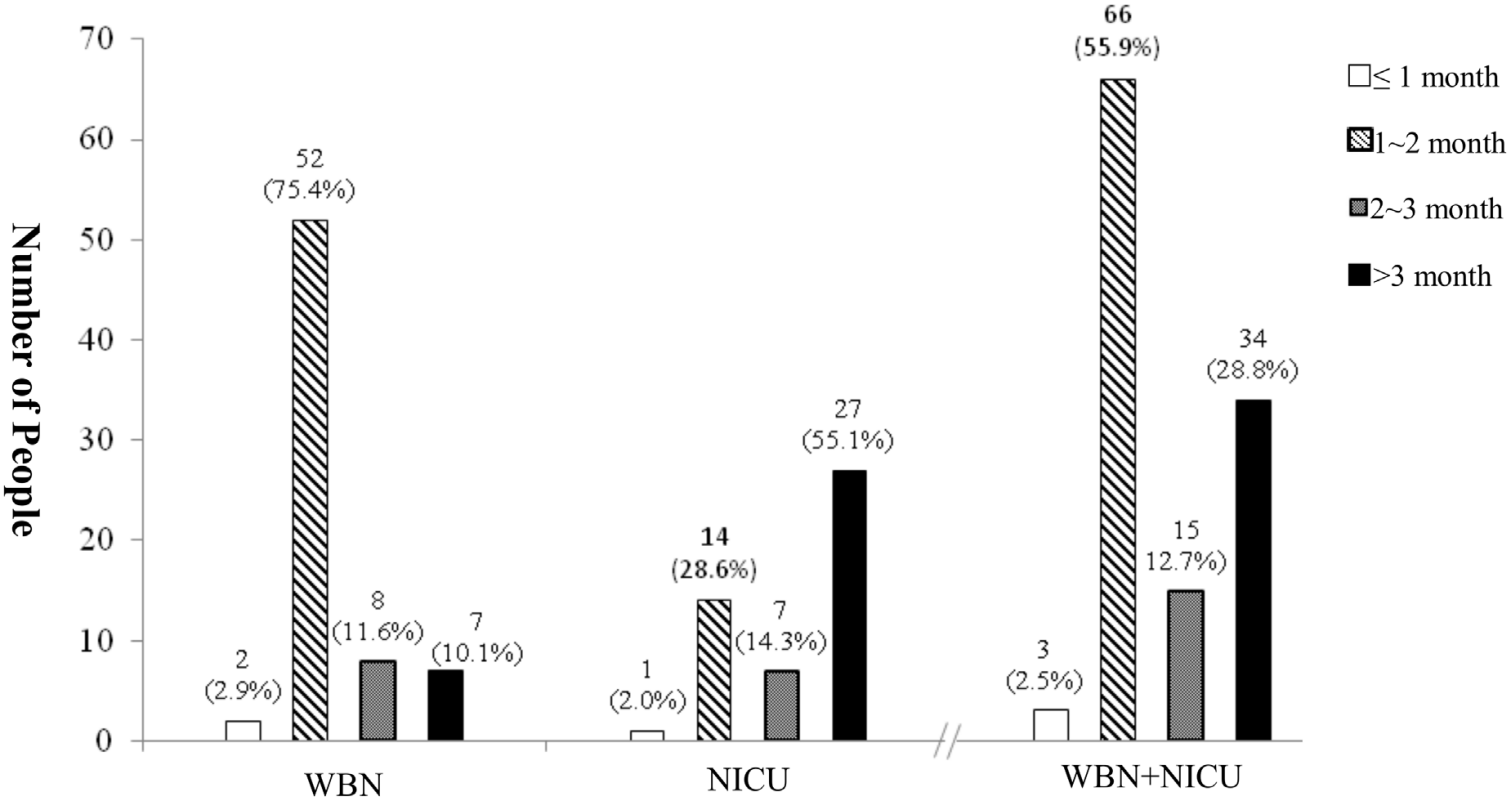
Numerical distribution of initial hearing diagnosis age in WBN (n = 69) and NICU (n = 49) groups.

A total of 118 babies (WBN, 69; NICU, 49) underwent complete diagnostic hearing assessment after being referred for hearing screening(Fig 3). In the WBN group, the diagnostic age of follow up was <1 month in 2 babies, 1–2 months in 52 babies, 2–3 months in 8 babies, and >3 months in 7 babies. In the NICU group, the diagnostic age of follow-up was <1 month in 1 baby, 1–2 months in 14 babies, 2–3 months in 7 babies, and >3 months in 27 babies. For the 118 babies who underwent diagnostic follow-up, the average age of diagnostic hearing assessment was 2.7 months; the age was 1.9 and 3.8 months in the WBN and NICU groups, respectively, exhibiting a significant difference (F = 11.697, p = 0.001; t = −4.066, p = 0.000). It revealed NICU babies received later hearing confirmed diagnosis due to later initial screening.

**Fig 4:**
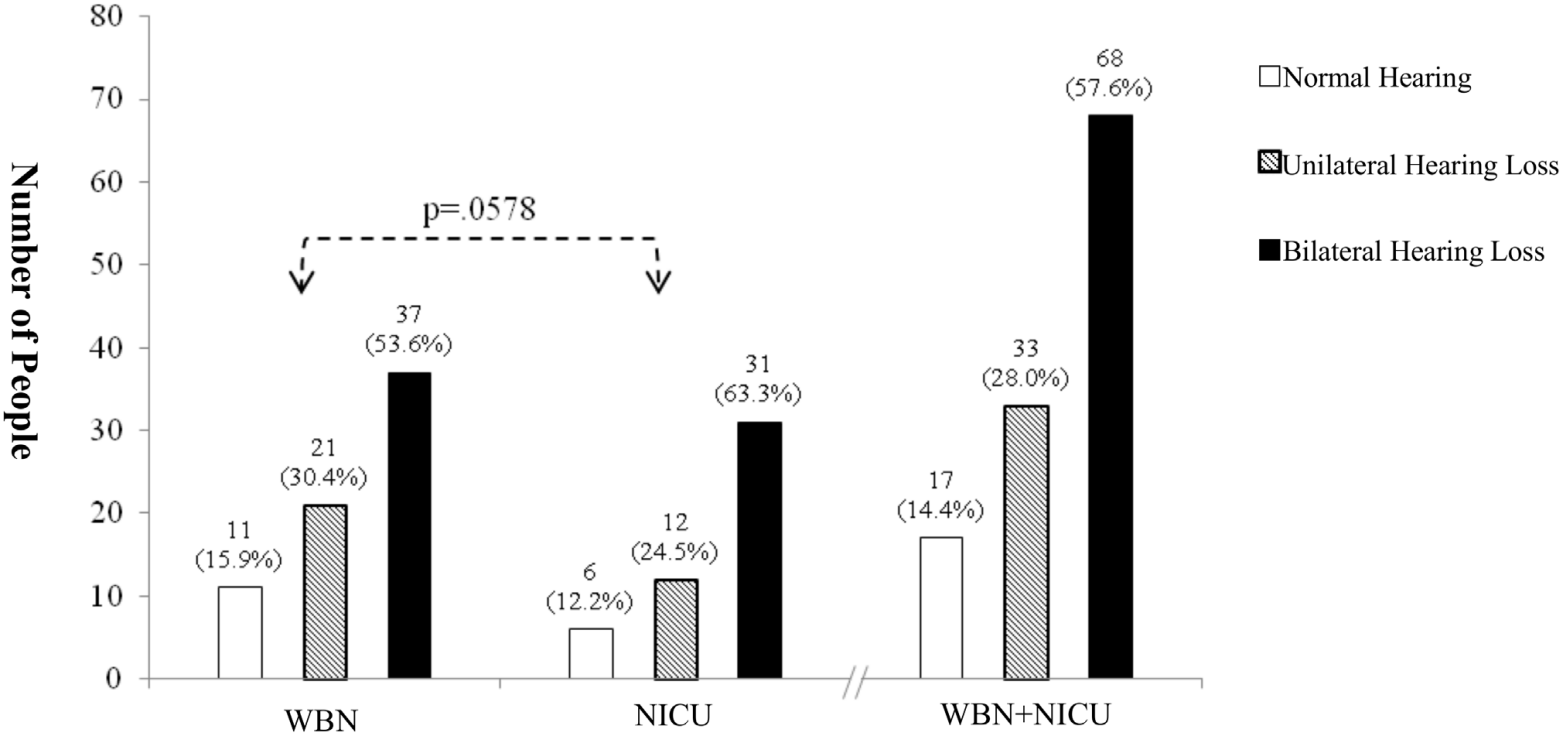
Numerical distribution of hearing diagnosis in WBN (n = 69) and NICU (n = 49) groups. There was no significant difference in the follow-up diagnosis results between the WBN and NICU groups (X2 = 1.096 (df = 2, p = 0.0578), p > 0.05).

Fig 4 shows that, of the follow-up 118 babies, 17 were identified to have normal hearing, 33 were diagnosed with unilateral HL, and 68 were diagnosed with bilateral HL after complete follow-up hearing examinations. The figure also shows that, of the 69 WBN follow-up babies, 11 were identified to have normal hearing, 21 were diagnosed with unilateral HL, and 37 were diagnosed with bilateral HL after complete hearing follow up; of the 49 NICU follow-up babies, 6 were identified to have normal hearing, 12 were diagnosed with unilateral HL, and 31 were diagnosed with bilateral HL. There was no significant difference in the follow-up diagnosis results between the WBN and NICU groups (Fig 4) (*X*^2^ = 1.096 (df = 2, p = 0.0578), p > 0.05).

**Fig 5:**
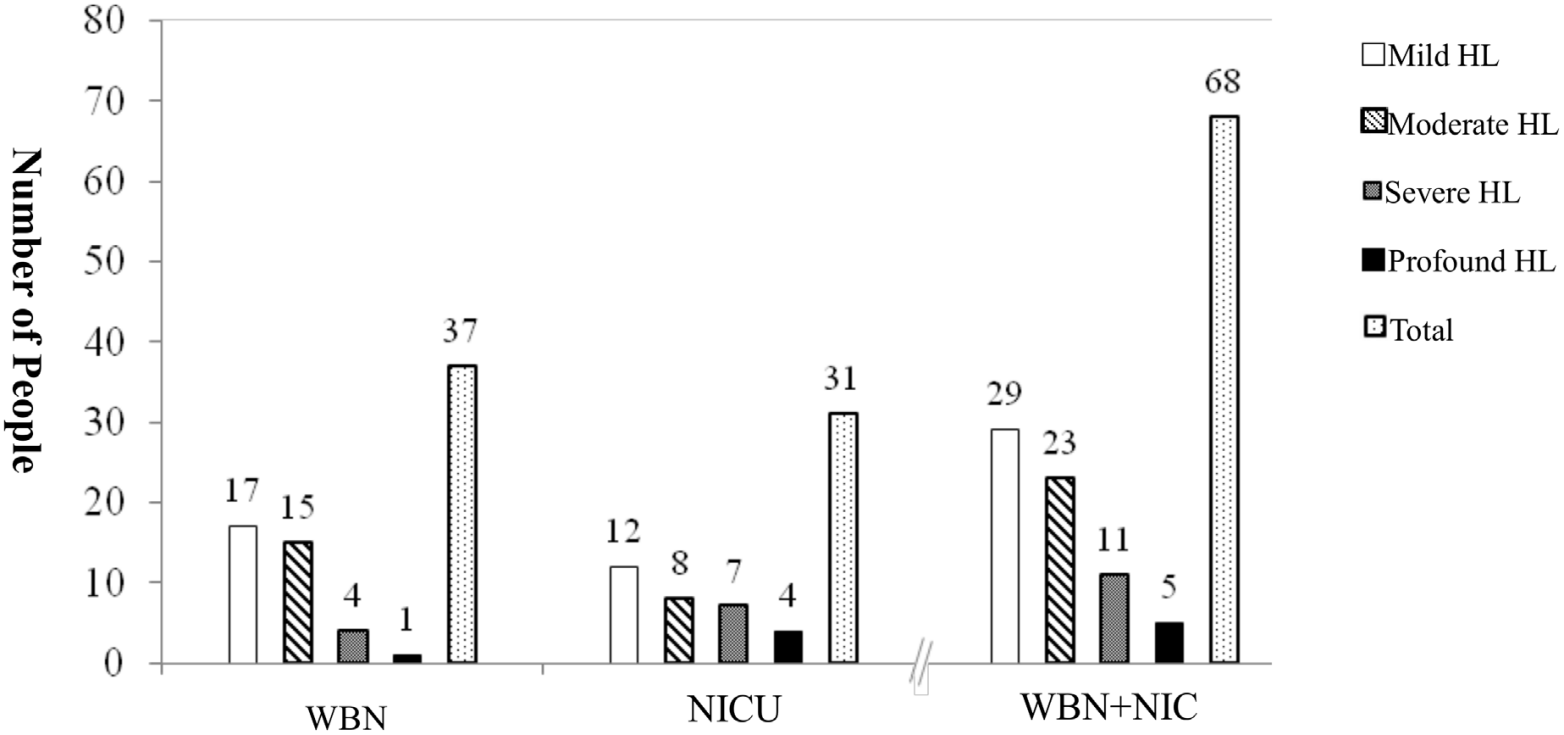
Population distribution of bilateral hearing impairment in WBN (n = 37) and NICU (n = 31) groups.

**Fig 6:**
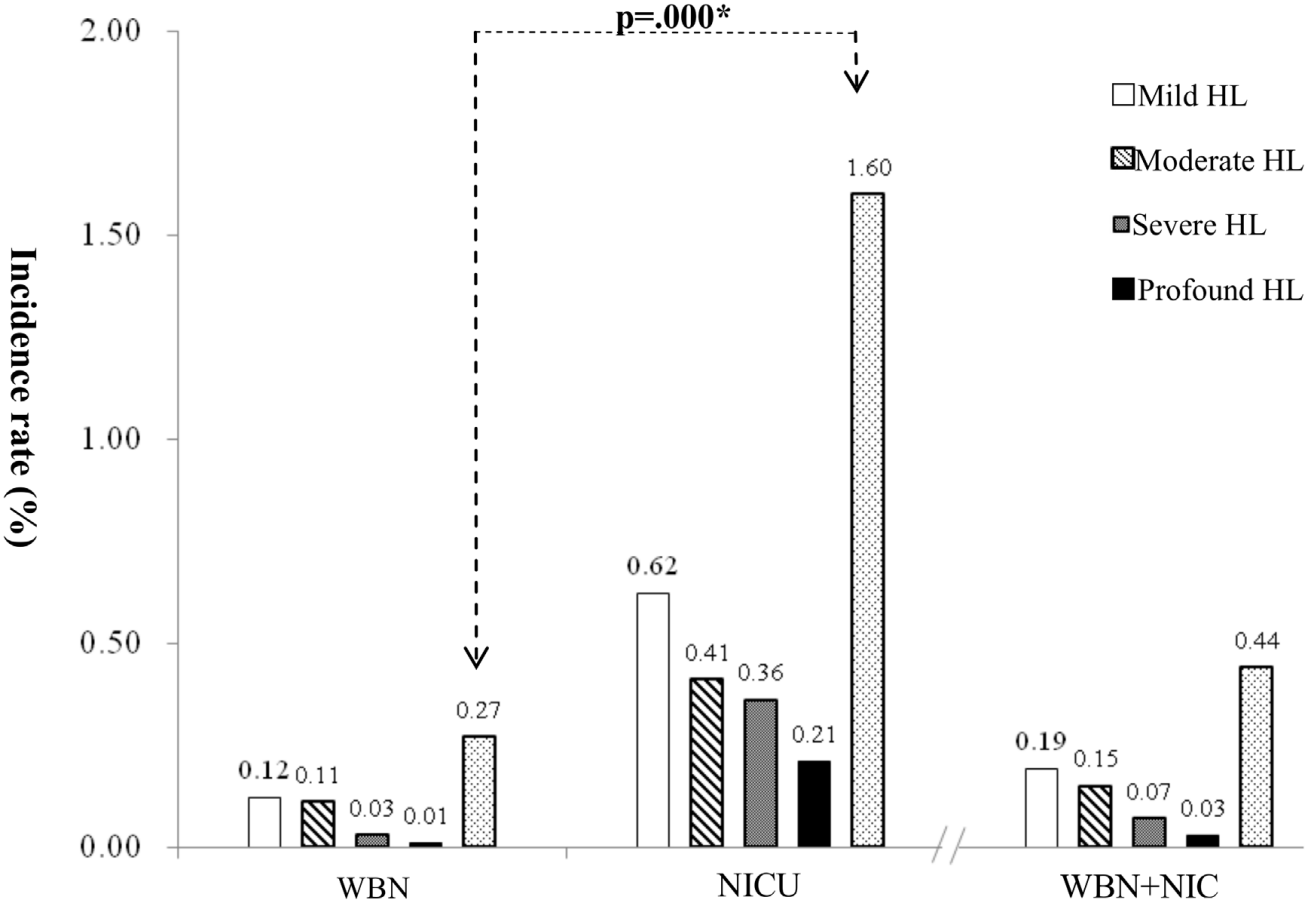
Incidence rate of bilateral hearing impairment in WBN (n = 37) and NICU (n = 31) groups. A comparison of the prevalence of congenital bilateral HL between the WBN and NICU groups showed a significant difference (X2 = 68.786 (df = 1, p = 0.000), p < 0.01).

Fig 5 show that 68 babies (WBN, 37; NICU, 31) were diagnosed with bilateral HL, and the age of diagnosis ranged between 4 and 37 months. Of these babies, 30 were diagnosed using ABR measurement and 38 were diagnosed using VRA. Regarding the degree of HL, 29 babies had mild HL (WBN, 17; NICU, 12), 33 babies had moderate HL (WBN, 15;NICU, 8), 11 babies had severe HL (WBN, 4;NICU, 7), and 5 babies had profound HL (WBN, 1; NICU, 4). Fig 6 shows that the prevalence of congenital bilateral HL were 0.44% in both groups, 0.27% in the WBN group, and 1.60% in the NICU group.

A comparison of the prevalence of congenital bilateral HL between the WBN and NICU groups showed a significant difference (*X*^2^ = 68.786 (df = 1, p = 0.000), p < 0.01).

## Discussion

This study included 15,624 (WBN, 13676; NICU, 1948) newborns screened for HL impairment using AABR. The hearing screening coverage rates were 99.8% (WBN, 13645/13676) and 99.6% (NICU, 1941/1948) in the WBN and NICU groups, respectively. In the WBN group, 31 babies did not undergo hearing screening (30 died; for 1, no test result was available); in the NICU group, 7 babies did not undergo hearing screening because of poor health conditions. The hearing screening coverage rate close to 100% observed in our study was also evident in another study conducted in Taiwan[9] and the U.S. [20]. The near 100% of coverage rate were achieved because of the implementation of the free national UNHS program in Taiwan in 2012 [9].

Fig 1 shows that the hearing screening referral rates were 0.7%, 2.8%, and 0.9% in the WBN group, NICU group, and both groups, respectively. Our study showed a higher hearing screening referral rate in the NICU group than in the WBN group, which results were similar to those observed in Dutch by van Dommelen[18] and van der Ploeg[21]. Connolly et al[13] reported that the high risk register factors for congenital deafness were observed in 1.2/1000 and 13.3/1000 babies in WBN and NICU groups, respectively. However, our referral rate in NICU was 2.8%, much lower than 4.9% by Mason Herrmann[17] NICU study in U.S., in which their refer rate was 4.9%(68/1401) using AABR and infants were screened at age of 2 days to 90 days, but no description of its average screening time. Our 2.8% referral rate was also much lower than 9.2% by van Dommelen [18] NICU study in Dutch, in which their refer rate was 9.2%(2933/32038) using AABR and 95.8% of infants were screened < 1 months, but without mention of its average screening time. Additionally, in one Brazil study[19], their refer rate was 4.1% using after twice AABR screening without statement of its average screening time. These three above studies seemed to that higher referral rate in NICU than us were observed in developed and developing countries. The more reasonable lower referral rate in our NICU study could be attributed to be a little late time set for hearing screening when infants were transferred to NBC with an average time of 13 Days, speculated to be late than the above three studies[17–19]. The second probable reason for lower referral rate in our NICU group could be attributed to our hearing screener with well experienced (> 10 years work) full-time nursery background.

Fig 2 shows that the total return-for-follow-up rate was 81.4% (118/145), and the rates for the WBN and NICU groups were 76.7% (69/90) and 89.1% (49/55), respectively. However, this difference was non-significant. Of the 21 babies who were lost to follow up in the WBN group, 18 babies with unilateral referral and 3 with bilateral referral. Upon being contacted for telephone counseling years later, the parents of the 3 babies with bilateral referral reported that their children had no hearing problems on the basis of their daily activity. Therefore, the reason for the lower follow-up rate in the WBN group could be the neglect of potential hearing problems because the babies had a unilateral hearing referral. Nursing personnel involved in UNHS should conduct more parents counseling to prevent loss to follow up for unilateral hearing examination in WBN babies. Of the 6 babies who were lost to follow up in the NICU group, 3 received a unilateral referral and 3 received a bilateral referral. Upon being contacted for telephone counseling years later, the parents of the 3 bilateral referral babies refused to talk about their children’s hearing behavior.

In the 118 babies who were followed up, the average age of diagnostic hearing assessment was 2.7 months in both groups, 1.9 months in the WBN group, and 3.8 months in the NICU group (Fig 3). The results showed a significant difference between the groups, indicating that there was a delay in the age of diagnostic hearing follow-up assessment in the NICU group(3.8 months) compared with the WBN group(1.9 months). In addition, 78.3% of referred babies in the WBN group received diagnostic hearing follow-up assessment at the age of <2 months, and 55.1% of the referred babies in the NICU group received this assessment at the age of >3 months (Fig 3). These results are similar to those observed by Mason and Herrmann[18]. The major reason for the delay of diagnostic hearing follow-up assessment in the NICU group was poor health conditions.

Fig 4 shows that, of the total 118 babies referred after the complete hearing diagnosis follow up, 17 had normal hearing, 33 had unilateral HL, and 68 had bilateral HL. Of the 69 WBN babies referred after the complete hearing follow up, 11 had normal hearing, 21 had unilateral HL, and 37 had bilateral HL. Of the 49 NICU babies referred after the complete hearing follow up, 6 had normal hearing, 12 had unilateral HL, and 31 had bilateral HL. Of the 33 babies with unilateral HL, 21 and 12 were in the WBN and NICU groups, respectively. Of the 68 babies with bilateral HL, 37 and 31 were in the WBN and NICU groups, respectively.

The average age of confirmed hearing diagnosis in 68 babies with bilateral HL was 9.8 months (4–36 months). In our study, bilateral asymmetrical HL was considered to occur when the difference in the hearing threshold between the ears was >15 dB HL. The incidence of bilateral asymmetrical HL was higher in the NICU group than in the WBN group, although it showed no significant difference between the two groups.

Fig 5 shows the distribution of the degrees of HL in the 68 babies who were confirmed to have bilateral HL. The incidence of mild to moderate HL was higher in the WBN group than in the NICU group. Conversely, the incidence of severe to profound HL was higher in the NICU group than in the WBN group. The greater distribution of cases of moderate and profound bilateral HL in our study was consistent with the results of Mason and Herrmann [18].

The prevalence of bilateral congenital HL among the babies in both groups was 0.44% (0.27% in WBN and 1.6% in NICU groups, respectively), which is higher than that in our previous study (0.13%–0.16%)[8,11]. These two our results were different because, in this study, hearing screening was performed in both the WBN and NICU groups, but only in WBN babies were screened in our previous study[8,11]. In our study, the prevalence of bilateral congenital HL among babies in the WBN group was 0.27% (mild 0.12% vs. moderate–profound HL 0.15%), which is slightly higher than that of 0.07%–0.16% reported by studies conducted in Dutch[20], or U.S. [18]. The prevalence of bilateral congenital HL among the NICU group babies in our study was 1.6% (mild 0.62% vs. moderate–profound HL 0.92%); this prevalence rate is consistent with 1.7% of Van Dommelen study in Dutch[17] and that observed by Seewald and Tharpe[22], who reported that the prevalence of bilateral congenital deafness in NICU babies was approximately 0.8%−2.0%, which was 10 times higher than that in WBN babies.

The limitation of our study was that it was a cohort study conducted at a single medical center; in the future, studies should examine babies from multiple centers.

## Conclusion

**Fig 7:**
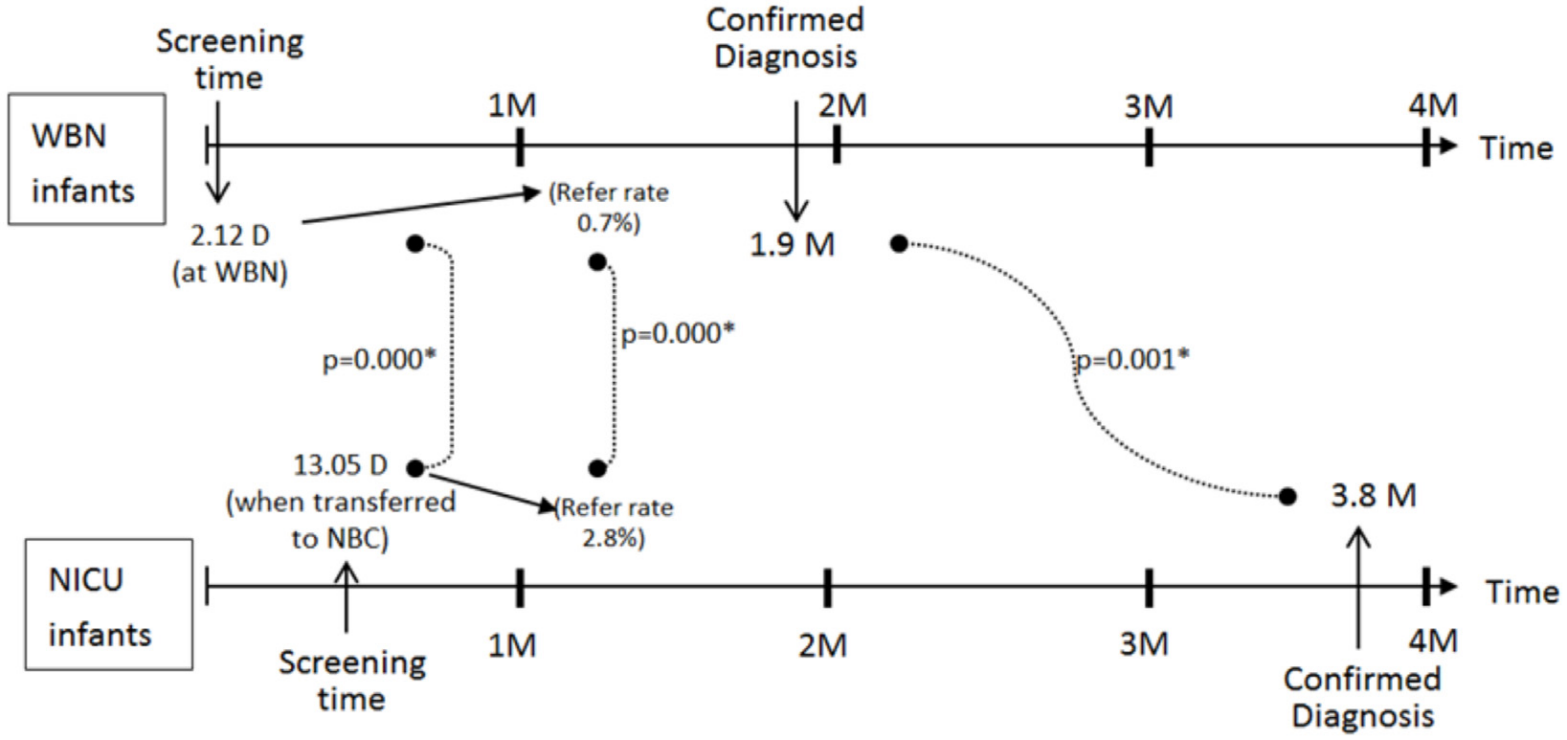
A time algorithm for newborn hearing screening set for WBN and NICU infants.

The hearing screening rates in the WBN and NICU groups were 99.8% and 99.6%, respectively, with no significant difference. The referral rates in the WBN and NICU groups were 0.7% and 2.8%, respectively, with a significant difference. The lower 2.8% hearing referral rate in our NICU group than before studies could be attributed to a little late time set for hearing screening or our hearing screener with experienced full-time nursery background. The diagnostic follow-up rates in the WBN and NICU groups were 76.7% and 89.1%, respectively, without significant difference. The average initial diagnostic ages in the WBN and NICU groups were 1.9 months and 3.8 months, respectively, with a significant difference. The prevalence rates of congenital bilateral HL in the WBN and NICU groups were 0.27% and 1.6%, respectively, with a significant difference. Importantly, we also concluded a well hearing screening time algorithm set at about 2 weeks in NICU could reduce referral rate (Fig 7).
